# Possible Genotoxic Effects of Post-Harvest Fungicides Applied on Citrus Peels: Imazalil, Pyrimethanil, Thiabendazole and Their Mixtures

**DOI:** 10.3390/foods14071264

**Published:** 2025-04-03

**Authors:** Boglárka Bernadett Tisza, Luca Járomi, Judit Háhn, Bálint Bérczi, Andrea Horváth-Sarródi, Andrea Gubicskóné Kisbenedek, Gellért Gerencsér

**Affiliations:** 1 Doctoral School of Health Sciences, Faculty of Health Sciences, University of Pécs, 7621 Pécs, Hungary; boglarka.tisza@etk.pte.hu; 2 Department of Public Health Medicine, Medical School, University of Pecs, 7624 Pécs, Hungary; berczi.balint84@gmail.com (B.B.); andrea.sarrodi@aok.pte.hu (A.H.-S.); gellert.gerencser@gmail.com (G.G.); 3 Department of Environmental Safety, Institute of Aquaculture and Environmental Safety, Hungarian University of Agriculture and Life Sciences, 2100 Gödöllő, Hungary; hahn.judit@uni-mate.hu; 4 Institute of Nutrition Sciences and Dietetics, Faculty of Health Sciences, University of Pécs, 7621 Pécs, Hungary; andrea@etk.pte.hu; 5 Preclinical Research Center, Medical School, University of Pécs, 7624 Pécs, Hungary

**Keywords:** citrus, peels, fungicides, imazalil, thiabendazole, pyrimethanil, comet assay, DNA fragmentation, mutations

## Abstract

Post-harvest fungicides are frequently used on citrus peels to reduce post-harvest fungal contamination during the storage and transport of products. Despite these positive effects, fungicides can pose health risks to consumers. The aim of our study was to perform a genotoxicological risk assessment of imazalil, pyrimethanil, thiabendazole and their mixtures used as post-harvest treatments. A *Salmonella* mutagenicity Ames test and comet assay were performed to detect reverse mutation and assess DNA damage. Base-pair, frameshift mutations and metabolic activity were analyzed using the Ames test. In the comet assay, lymphocytes were treated with fungicides for 4 and 24 h. Thiabendazole was found to induce both frameshift and base-pair mutations in the Ames test despite the mutagenicity of both imzalil and pyrimethanil (*p* < 0.05). DNA-strand breaks were observed in lymphocytes, mainly with dimethyl-sulfoxide solvent fungicides (*p* < 0.05). The long-term exposure and consumption of fruits and vegetables treated with fungicides can increase the risks of developing genotoxic tumors. Our findings raise new questions about the health risks of fungicides and their mixtures to consumers. Further investigations are essential to explore the genotoxicological effects of fungicides on citrus peels.

## 1. Introduction

In food production today, agriculture aims to increase yields, improve crop quality and extend the shelf life of food crops. All this relies on the widespread utilization of chemicals such as synthetic fungicides. Fruits undergoing synthetic pesticide treatment are still being offered for sale in the markets. Synthetic fungicides used in agriculture are highly effective in reducing post-harvest fungal mortality during the storage and transport of agricultural products [1]. To increase shelf-life, a large variety of post-harvest treatments with fungicides are employed in agriculture, such as a wax mixture by spraying, an aqueous solution by dipping method and an aqueous solution by thin film method [2]. The peels are treated with shellac and wax, which can contribute to the formation of a protective layer on the peels that can help to retain pesticides on the surface [3]. The weather conditions in the fields (rain, wind, UV radiation, temperature) and the storage time of harvested crops can cause the degradation of fungicides, and plant metabolism can also eliminate or degrade pesticides [4]. In everyday life, the residual pesticide content of citrus fruits has always been a cause for concern, as food safety waiting periods required in the post-harvest handling of fruits differ from those applied in arable farming, where the aim is to ensure the timely delivery of crops to consumers [5,6]. These chemicals can contaminate soil, water and air, threatening biodiversity and harming ecosystems. In recent decades, organic fruit has become increasingly popular and accessible to consumers, in line with informed purchasing decisions. Organic food has been shown to contribute to sustainability through environmentally friendly production; it can reduce pollution without the use of fertilizers and it is beneficial for pest control [7,8]. However, there is still a lack of information about the harmful effects of fungicides used in agricultural production today. Besides vegetables, fruits such as citrus are also widely treated with a variety of fungicides against several pathogenic fungi that can cause damage to the organism [9], but their precise molecular and biological mechanisms and long-term effects are not yet known.

Synthetic fungicides, such as imazalil, thiabendazole and pyrimethanil, are popular postharvest fungicides for the control of green and blue molds on citrus fruits [10]. The American Environmental Protection Agency (EPA) has classified imazalil as a probable carcinogen [11], and its adverse effects have already been explored [12,13]. Imazalil is widely used in crop protection for a variety of treatments. Imazalil has been shown to have the highest acute reference dose percentage both among children (max ARfD 1087%) and adults (max ARfD 251%). It was also detected at the highest pesticide residue concentrations in oranges (4.10–0.096 mg/kg), clementine oranges (2.40 mg/kg), pomelo fruit (0.02 mg/kg), lemons (1.53–0.33 mg/kg) and grapefruit (0.37–0.14 mg/kg) [14].

Maximum residual level (MRL) is regulated by the European Union and defined separately for each product and food. The MRL was the following for each citrus fungicide: 7 mg/kg for thiabendazole (Reg. (EU) 2024/1342), 4–5 mg/kg for imazalil (Reg. (EU) 2020/856) and 4–5 mg/kg for pyrimethanil (Reg. (EU) 2020/856) [15].

Pyrimethanil is an environmental pollutant with possible cytotoxic effects, and it has a low acute toxicity to humans [16,17]. Pyrimethanil has also been shown to be ecotoxic [18,19,20]. Based on EPA’s categorization of thiabendazole as a probable human carcinogen at high doses, further toxicological studies have identified carcinogenic, hepatotoxic, ecotoxic and genotoxic effects [21,22,23]. Consumers are also exposed to fungicides that may have adverse health effects, with residues detectable and measurable in fruit peel and pulp [24,25]. The peel of citrus fruits is often used during the preparation of food, jams, cakes, desserts and beverages, raising concerns that pesticide residues may be present in higher proportions in the peel than in other parts of the fruit [26,27]. In samples of mandarins, imazalil showed 58% migration from the peel into the albedo and 6% migration into the flesh. The highest concentration of imazalil was detected in orange samples (4468 μg/kg), and imazalil was the most frequently observed pesticide [28]. Fungicides and fungicide mixtures are associated with a range of toxicological effects, including genetic damage. Pesticides have also been found in combination, and the effects of mixtures may produce synergistic effects, which are also well documented. Genotoxic risk assessment of fungicides and their combinations is essential for public health and safety reasons. With regard to the consumption of fruit and vegetables, synthetic pesticide contamination is the main food safety concern for consumers [29].

This preliminary study aims to perform a genotoxic risk assessment and to evaluate the mutagenic effects of fungicides such as imazalil, thiabendazole and pyrimethanil and their joint effects in combination.

## 2. Materials and Methods

### 2.1. Fungicides and Application

Fungicides applied in agriculture (according to the description and dosage of products), imazalil (IMA) at 6.8 mg/100 mL, thiabendazole (TBZ) at 5 mg/100 mL and pyrimethanil (PYR) at 5 mg/100 mL concentration (Merck-Sigma-Aldrich, Budapest, Hungary) were each dissolved in a distilled water and dimethyl sulfoxide (DMSO) solvent as a stock solution. The mixture of fungicides was measured in a ratio of 1:1:1; we prepared both aqueous and DMSO solvent mixtures.

### 2.2. Ames Test

The *Salmonella* Ames test is a mutagenicity test to detect reverse mutation. The aim was to start the research with a prokaryotic organism. This method is designed to test several chemical substances that may cause genetic damage. The *Salmonella typhimurium* TA 98 strain was used to detect frameshift mutation, while the TA 100 strain was considered adequate for base-pair mutation analysis. *Salmonella* TA 98 and *Salmonella* TA 100 bacteria strains (Trinova Biochem GmbH, Gießen, Germany) were inoculated in Oxoid nutrient broth (Merck-Sigma-Aldrich, Budapest, Hungary) to reach a bacterial count of 10^8^/mL. We added and layered the following to the minimal medium: 2 mL of top agar (Merck-Sigma-Aldrich, Budapest, Hungary), pesticides (20.4 μg/plate imazalil, 15 μg/plate for both pyrimethanil and thiabendazole; 16.8 μg/plate of fungicide samples’ mixture, then, finally, twice as much of the mentioned doses) and 100 µL of bacteria. We also evaluated the indirect mutagenic effect of the samples by adding an enzyme. We added 500 µL of enzyme S9 (locally prepared) mixed with cofactors. Mutagenic effects were observed both directly (S9−) and indirectly (S9+). Negative controls (that contained DMSO, aqueous solvent) and positive controls were also used in this study. The tests and measurements were performed in triplicate. The bacteria were incubated at 37 °C for 48 h. The revertant colonies that we calculated are indicators of a mutagenic effect [30].

### 2.3. Comet Assay

The in vitro comet assay is a sensitive, reliable method to assess DNA damage in the case of any type of eukaryotic cell [31]. We used the alkaline version of the comet assay, which is suitable for detecting both single-strand and double-strand DNA breaks. This investigation involved human cells (one subject), and this study was approved by the Hungarian Medical Research Council ((13709-8/2023/EÜIG), 2023.).

#### 2.3.1. Gel Slide Preparation

We applied three-layer gels on fully frosted microscopic slides. The first layer (150 µL) contained an NMA (normal melting point agarose gel, 0.5% (Merck-Sigma-Aldrich, Budapest, Hungary)) in 75 µL and contained the fungicides in 75 µL. The middle layer (75 µL) was LMPA (low melting point agarose, 0.5% (Merck-Sigma-Aldrich, Budapest, Hungary)), to which peripheral lymphocytes were added in a volume of 10 µL (approximately 10,000 cells, one drop of blood from fingertip). The third agarose layer (75 µL) was once again an LMPA, containing no test sample or human blood. The incubation was performed in a dark wet chamber at 37 °C for a duration of a minimum of 60 min; the gel slides were placed in a lysing solution (15 sodium sarcosinate, 2.5 NaCl, 100 mM Na_2_-EDTA, 1% Triton X-100, 10% DMSO and 10 mM Tris (Merck-Sigma-Aldrich, Budapest, Hungary)). The slides were immersed in an electrophoresis buffer solution containing 200 mM EDTA (Merck-Sigma-Aldrich, Budapest, Hungary) and 10 mM NaOH (Molar Chemicals Kft, Budapest, Hungary) at pH 10 for 20 min [32]. Electrophoresis (Hoefer, San Francisco, CA, USA) was performed in a dark room for 40 min at 132 mA and 0.46 V/cm. The slides were neutralized three times for 5 min with a neutralization buffer solution (0.4 M Tris) and stained with 50 μL ethidium bromide (Merck-Sigma-Aldrich, Budapest, Hungary). A minimum of 50 nuclei were analyzed on each slide using the Comet Assay IV Imaging Software, Version 4.3.1 (400×, Perceptive Instruments Ltd., Bury St. Edmunds, UK). The DNA damage was evaluated by assessing the tail moment product of the DNA [33]. Negative controls, such as an aqueous solvent and DMSO, and positive controls were also used in this study.

#### 2.3.2. Cell Culture Preparation

For the cell culture preparation, 5.3 mL of RPMI 1640 medium containing L-glutamine (Merck-Sigma-Aldrich, Budapest, Hungary), 0.5 mL of fetal bovine serum (Merck-Sigma-Aldrich, Budapest, Hungary) and 0.05 mL of antibiotic (Penicillin-Streptomycin, Merck-Sigma-Aldrich, Budapest, Hungary) were mixed in each well (6-well plate); DMSO and aqueous fungicide samples (imazalil, pyrimethanil, thiabendazole and a mixture of the three fungicides) in different concentrations (Table 1) were measured from the stock solution and added to the mixed medium; then, 400 μL blood was added to each sample in the wells. The plates were placed in a humified incubator for 4 and 24 h under the conditions at 37 °C with 5% CO_2_. The cells were centrifuged at low speed (1000 rpm (150× *g*) for 5 min). The supernatant was discarded and the residual culture was resuspended with 1 mL of physiological saline (B Braun, Melsungen, Germany) and centrifuged again. The cell culture was resuspended in 0.5 mL physiological saline. A total of 150 μL of normal melting point agarose (NMA) gel was dispensed on the slide and sealed by a cover slip for solidifying the agarose. As a second layer, 500 μL of low melting point agarose (LMPA) and 50 μL of treated lymphocyte cell culture with fungicides were used and 100 μL of this mixture was dispensed on top of the first layer of gel. As a third layer, 100 μL of low melting point agarose (LMPA) was added which no longer contained either the test sample or human blood. Once each layer was applied, a cover slip was placed on the still liquid gel to achieve uniform spreading of the gel. We followed the same protocol with electrophoresis for the cell culture as well as the gel slide preparation [33].

### 2.4. Statistical Analysis and Methods

To evaluate the results, a statistical data analysis was performed using IBM SPSS Statistics Version 26.0 for Windows (Armonk, NY, USA). An independent sample *t*-test was conducted for the analysis of comet assay data to assess genotoxic effects by comparing the samples to the control. For the Ames test, we also ran an independent sample *t*-test [34]. We presented the data as mean ± SD. All the measurements were performed as triplicates. Results were considered significant if *p* < 0.05.

## 3. Results

### 3.1. Ames Test Results

The Ames test is an in vitro bacterial bioassay in which we used *Salmonella* TA98 and TA100 bacteria in order to determine whether the applied chemical may cause a potential mutation directly and indirectly in the prokaryotic DNA. We performed the test using aqueous and DMSO solvent fungicides.

We observed a bacterial background lawn in the microscope, which means the pesticides were not cytotoxic to the test microorganism. Concerning the aqueous fungicides, we mostly detected base-pair mutations without the S9 enzyme (Table 2). A direct base-pair mutation and an indirect frameshift mutation were found to be caused by imazalil at both dosages (*p* < 0.05). The 15 and 30 μg/plate of thiabendazole induced a direct frameshift mutation and an indirect base-pair mutation (*p* < 0.05). The direct base-pair mutation was observed after the treatment of 15 μg/plate of pyrimethanil and 30 μg/plate of thiabendazole. Pyrimethanil also induced an indirect base-pair mutation at double dosage (*p* < 0.05). The combined samples were not found to induce mutagenic effects.

We observed significant differences in isolated cases after treating the cells with fungicides prepared in DMSO (Table 3). The background lawn was also detectable under the microscope. At a lower dosage (20.4 μg/plate), imazalil induced base-pair mutation directly, while a double dosage was conducive to indirect frameshift mutation (*p* < 0.05). Thiabendazole at 30 μg/plate was found to be mutagenic indirectly and directly, inducing a frameshift mutation; furthermore, at the 15 μg/plate dosage, a frameshift and base-pair mutation was observed both directly and indirectly (*p* < 0.05). As in the case of aqueous fungicide cocktails, the mixtures in DMSO did not indicate any mutations in the strains.

### 3.2. DNA Lesions Evaluated by the Comet Assay

The comet assay method was used to assess the genotoxic effects of each fungicide and its mixtures on the number of DNA lesions by evaluating the tail moment of each lymphocyte. The effects of different concentrations of fungicides (Table 1) were also tested. The gel slide preparation and cell culture methods were analyzed; furthermore, the impact of treatment duration (4 h and 24 h) on the cell culture was also evaluated. Significantly higher values of tail moments as compared to controls could indicate a genotoxic effect of fungicides.

**Figure 1:**
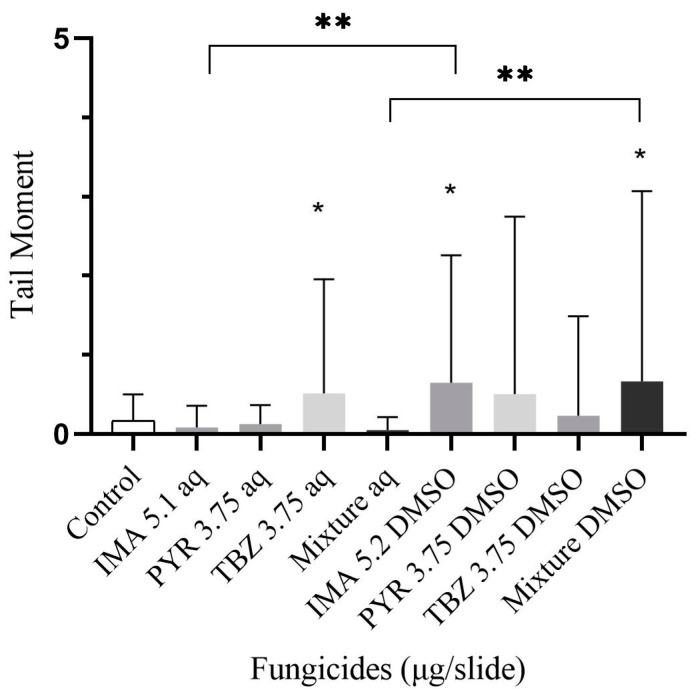
DNA lesions marked as tail moments with gel slide preparation method. Data were presented in mean ± SD. * Significant if *p* < 0.05. ** Significant differences between samples (*p* < 0.05). The mixture contains imazalil (1.7 μg/slide), pyrimethanil (1.25 μg/slide) and thiabendazole (1.25 μg/slide).

Aqueous thiabendazole, like imazalil and the mixtures in DMSO, showed genotoxicity (*p* < 0.05). Thiabendazole in aqueous solution appeared to be more genotoxic compared to the mixture (*p* < 0.05). The highest tail moments can be observed for imazalil and the combined fungicides in the DMSO solvent. Notably, in the DMSO solvent, imazalil and the mixture showed significantly higher tail moment values compared to the aqueous samples (*p* < 0.05) (Figure 1).

**Figure 2:**
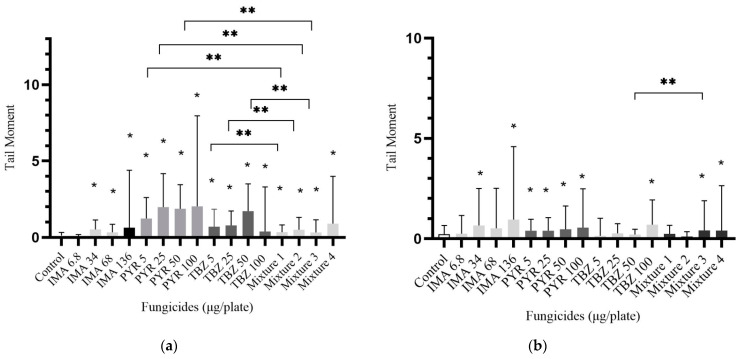
DNA lesions of lymphocyte cell culture. (**a**) Treatment with fungicides in aqueous solvent for 4 h; (**b**) cells treated with fungicides in aqueous solvent with a 24 h long treatment. Data are presented in mean ± SD. Data were significant if *p* < 0.05: * compared to the control; ** compared to the mixture. The black column shows where we measured less than 50 nuclei. Mixtures contain imazalil + pyrimethanil + thiabendazole as 2.27 + 1.67 + 1.67 µg/plate in Mixture 1; 11.35 + 9.35 + 8.35 µg/plate in Mixture 2; 22.7 + 16.7 + 16.7 µg/plate in Mixture 3 and 45.4 + 33.4 + 33.4 µg/plate in Mixture 4.

The levels of DNA lesions (tail moment) in lymphocytes after 4 h treatment of cell cultures showed a significant increase with aqueous imazalil (*p* < 0.05), pyrimethanil (*p* < 0.001), thiabendazole (*p* < 0.05) and the mixture (*p* < 0.05) (Figure 2a). Lymphocytes treated with 136 μg/plate imazalil had less than 50 cells on the plate, which is marked with a black column. No genotoxic effect was observed at 6.8 μg imazalil (*p* > 0.05). Regarding pure fungicides, pyrimethanil and thiabendazole at 5, 25 and 50 μg/plate concentrations showed significantly higher DNA damage relative to the mixture. Treatment with from 6.8 to 136 μg/plate resulted in an eightfold increase in tail moments for imazalil, with the mixture showing a 4.5-fold increase. Thiabendazole resulted in a fourfold increase, while pyrimethanil resulted in a 1.7-fold increase, which represents the smallest difference.

Genotoxicity was detectable in the case of all pyrimethanil concentrations in the course of the 24 h aqueous sample treatment (*p* < 0.05) (Figure 2b). The highest doses of imazalil, thiabendazole, pyrimethanil and the mixture were genotoxic in most cases (*p* < 0.001). Genotoxicity was confirmed for every pyrimethanil sample (*p* < 0.001). A 4.5-fold increase was observed for imazalil, a 1.25-fold elevation for pyrimethanil, a 1.5-fold elevation for thiabendazole, and, finally, a 3.5-fold increase was noted in the case of the mixture. The mixture with the parallel dosage caused significantly higher DNA damage compared to the 50 µg/plate dosage of thiabendazole (*p* < 0.05).

**Figure 3:**
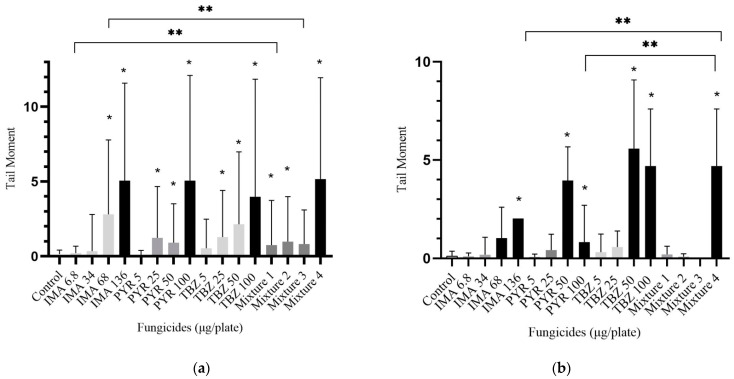
DNA lesions of lymphocyte cell culture. (**a**) Treatment with fungicides in DMSO solvent for 4 h; (**b**) cells treated with fungicides in DMSO solvent with a 24 h long treatment. Data presented as mean ± SD. Data were significant if *p* < 0.05: * compared to the control; ** compared to the mixture. Mixtures also contain imazalil + pyrimethanil + thiabendazole as 2.27 + 1.67 + 1.67 µg/plate in Mixture 1; 11.35 + 9.35 + 8.35 µg/plate in Mixture 2; 22.7 + 16.7 + 16.7 µg/plate in Mixture 3 and 45.4 + 33.4 + 33.4 µg/plate in Mixture 4. Black columns indicate measurements of less than 50 nuclei.

The 4 h treatment in the DMSO samples yielded the following results: significant differences in DNA fragmentation were detected for the 68 and 136 µg/plate treatments of imazalil (Figure 3a). The 6.8 and 34 µg/plate values showed no genotoxic effect at all. Pyrimethanil and thiabendazole were found to be genotoxic under treatments at 25, 50 and 100 µg/plate (*p* < 0.05). Notably, pyrimethanil treatment at concentrations of from 5 to 100 µg/plate resulted in a 72-fold increase, and imazalil treatment at concentrations of from 6.8 to 136 µg/plate resulted in a 26-fold increase (*p* < 0.001). The mixture showed a significantly higher genotoxicity in the first two lowest concentrations’ treatments compared to imazalil (*p* < 0.05). Less than 50 nuclei were recorded under the highest treatments of concentration, which indicates cytotoxicity.

In seven cases, we measured less than 50 tail moments due to lymphocyte apoptosis after a 24 h treatment with fungicides dissolved in dimethyl sulfoxide solvent (columns are highlighted with black color) (Figure 3b). Imazalil was genotoxic only at the highest concentration (136 μg/plate) (*p* < 0.001), with only one cell detectable (2.02 tail moment) and others remaining apoptotic on the slides. For pyrimethanil at the 50 and 100 μg/plate concentrations, we detected genotoxicity but still found four detectable lymphocytes with a tail moment of 0.83 ± 1.8 at the 100 μg/plate concentration. In the course of the investigation, the highest tail moment was recorded under the 24 h treatment of thiabendazole (5.5 ± 3.4 tail moment), where the samples were dissolved in DMSO solvent. During the combined treatment of the third highest concentration (22.7 + 16.7 + 16.7 μg/plate), no measurable lymphocyte cell was detected under the microscope. Genotoxicity (*p* < 0.05) was confirmed at the highest concentration of mixed pesticides, although less than 50 nuclei were enumerated. The highest mixture dosage indicated significantly higher DNA damage as compared with imazalil and pyrimethanil (*p* < 0.001).

## 4. Discussion

Synthetic fungicides are reliable in controlling postharvest citrus diseases; their application, however, may result in resistance to fungal infection [35]. Several questions have been raised concerning the impacts of fungicides applied on citrus peels. These pesticides are viewed as toxic substances and human health hazards [36].

In the present preliminary study, we performed a genotoxic risk assessment of imazalil, pyrimethanil, thiabendazole and their mixture. This work illustrates the differences in mutagenic effects and the rate of DNA damage between mixtures and individual fungicides. According to our best knowledge, the risk assessment of imazalil, pyrimethanil, thiabendazole and their mixtures has, so far, received scant scholarly attention. These fungicides are widely used on citrus peels, which raises new questions about the risks of fungicides to both consumers and workers who work with them daily. Our findings demonstrate that the observed pesticides have potential genotoxic effects.

The Ames test relies on a prokaryotic system to detect reverse mutation. Ilyushina et al. [37] conducted research to evaluate the genotoxic effect of imazalil, imidacloprid and tebuconazole, both separately and as a mixture. The bacteria were treated with 1.25 mg/plate imazalil and were applied as a mixture in 0.05, 0.16, 0.5, 1.6 and 2.5 mg/plate concentrations. They found the pesticides to be non-genotoxic separately and as a mixture by applying *Salmonella typhimurium* strains TA97, TA98, TA1535, TA100 and TA102. The results show no evidence of mutagenic effects including or excluding the S9 enzyme. In a similar vein, our study has demonstrated that the mixture of fungicides is not conducive to reverse mutation despite the presence of genotoxicity at 20.4 and 40.8 μg/plate concentrations, respectively. Thiabendazole is shown to have no genotoxic effects when tested with the same strains of *Salmonella typhimurium* at 0.05, 0.16, 0.5, 1.6 and 5.0 mg/plate concentrations [38]. However, with thiabendazole treatment, they also observed a decrease in revertant colonies with increasing concentration, which may indicate toxicity. Regarding the effects of DMSO solvent thiabendazole in our study, the number of revertant colonies was higher. The mutagenic effect was present under both concentrations and was markedly high for thiabendazole in DMSO. The applied solvent may contribute to these results. We wanted to ensure that the fungicides could penetrate cells by DMSO. All in all, we detected mutagenicity at concentrations three orders of magnitude lower compared to the mentioned studies.

Kirkland et al. [39] performed a database analysis of pesticides’ positive results for comet endpoints, micronucleus and further genotoxic tests. Thiabendazole was found to be equivocal in the Ames test and also in the micronucleus test, with their effects not being clearly detectable in these experiments. Thiabendazole was reported to be positive in the mouse-lymphoma assay; this fungicide was characterized as its micronucleus positive results can be detected in a narrow concentration range and the toxicity profile was steep. Additionally, mutagenicity and DNA-damaging activity were investigated through a 10 min thiabendazole (50–400 μg/mL) treatment with UVA irradiation. Frameshift mutations and base-substitution mutations were discovered by the Ames test; this fungicide was characterized as an aneuploidy-inducing spindle poison [40]. In addition to causing damage in human lymphoblastoid cells, which were detected by the comet assay, this fungicide has been shown to trigger a significant increase in micronuclei.

The comet assay is a sensitive method to assess the genotoxic effect of a potentially harmful substance by measuring the rates of DNA-strand breaks. Two techniques of application were also compared, namely, the gel slide and cell culture methods. Differences in the treatment time were noted stemming from modeling 4- and 24-hour long exposures. The cell culture technique yielded more prominent tail moment values as compared with gel slide preparation. This can be attributed to the different techniques; in gel slide preparation the cells were more isolated in the gel medium, while the cell culture method involved direct treatment with fungicides.

Đikić et al. [41] evaluated the impact of imazalil and pesticide mixtures using a comet assay. DNA strand breaks were analyzed on mice hepatocytes. In this experiment, mice consumed 10 mg kg^–1^ of imazalil and cypermethrin and 20 mg kg^–1^ of carbendazim and their mixtures for a period of 28 days. Imazalil and thiabendazole both showed cytotoxicity on liver cells which is also suggested as an apoptotic effect [42], in line with the findings of our research. Likewise, imazalil was reported to be genotoxic to the aquatic environment [43].

Vindas and co-workers evaluated the genotoxic effect of thiabendazole and imazalil at 25, 50, 75 and 100 µg/mL concentrations [44]. Imazalil caused significant DNA damage and was reported to be genotoxic in a dose-dependent way, as corroborated by other studies [45,46]. In a similar vein, our study has demonstrated a dose-dependent increase in tail moments in human lymphocytes. Thiabendazole at 200 mg/kg caused DNA damage in mouse stomach, liver, lung, brain, kidney, bone marrow and bladder cells, which echoes our results [47]. In our research, we also detected DNA damage in lymphocytes that were treated with thiabendazole. The literature review points to a dearth of studies on pyrimethanil, although it has been shown by the European Food Safety Authority to be unlikely to be genotoxic [48].

The FAO (Food and Agriculture Organization of the United Nations) defined the ADI (acceptable daily intake) as 0.03 mg/kg bw (body weight) for imazalil. The ADI of thiabendazole was defined as 0.1 mg/kg bw, and, finally, for pyrimethanil, as 0.2 mg/kg bw [49]. Compared to the mentioned intake values—after a 4 h long treatment—we detected the genotoxicity of imazalil at a concentration of 34 µg/plate. Aqueous thiabendazole and pyrimethanil seemed to be genotoxic at a concentration of 5 µg/plate. Overall, limited knowledge is available to date on the long-term effects of fungicide dietary exposure. Based on the results of our preliminary research, additional studies are required to explore the mechanisms of fungicides and their joint effect.

Summarizing our results, imazalil, thiabendazole and pyrimethanil could induce point mutations directly and indirectly in various ways, depending on the test conditions. All three fungicides tested have been found to cause primary DNA damage under different exposure conditions. The results of our research seek to raise awareness of the potential health risks of fungicides, such as malignant tumors, developmental disorders and metabolic diseases.

Among the cell cultures, we have found several apoptotic lymphocytes at the highest exposure dosages, as demonstrated by the DMSO solvent samples, revealing higher tail moment values as well. In the case of the highest concentrations, exposure conditions potentially appeared to be apoptotic, hindering the measurement of all the nuclei parameters. Samples in DMSO induced higher tail moment values in contrast to the aqueous samples; this solvent allows the substance to penetrate the cells. This corroborates the idea that a significant amount of fungicides can be transported to the cell by amphipathic DMSO by virtue of their capacity to dissolve lipophilic molecules and increase membrane permeability [50]. Departing from this feature, as the next phase of our research, we seek to investigate the genotoxic effects of fungicides mixed with waxes and citrus oils.

Instead of the application of synthetic fungicides, the ultimate goal should be to create alternative techniques that can eventually replace them in order to protect consumer safety. This transition may lower health hazards and encourage more environmentally friendly agricultural methods.

## 5. Conclusions

Our study aimed to evaluate the genotoxic risks of imazalil, pyrimethanil, thiabendazole and their mixtures. The results confirm the importance of further research to gain more in-depth knowledge about pesticide residues, given the key significance of food safety for consumers. Recently, there has been a growing trend for adopting alternative bio-based agricultural practices, applying bio-fungicides and biological control, such as essential oils, plant extracts and chitosan, which are considered to be less toxic to humans, safe and environmentally friendly. Finally, sustainable practices in food production can contribute to a more resilient agricultural system, mitigating the impacts of climate change and fostering food security.

## Figures and Tables

**Table 1:** Concentrations of aqueous and DMSO fungicide samples used in the comet assay.

Concentration	Imazalil	Pyrimethanil	Thiabendazole	Mixture (IMA + PYR + TBZ)
Gel slide preparation μg/slide	5.1	3.75	3.75	1.7 + 1.25 + 1.25
Cell cultures μg/plate	6.8	5	5	2.27 + 1.67 + 1.67 (Mixture 1)
	34	25	25	11.35 + 9.35 + 8.35 (Mixture 2)
	68	50	50	22.7 + 16.7 + 16.7 (Mixture 3)
	126	100	100	45.4 + 33.4 + 33.4 (Mixture 4)

**Table 2:** Number of *Salmonella typhimurium* colonies (fungicides in aqueous solvent).

Aqueous Samples	Concentration μg/plate	TA98 (S9−)	TA98 (S9+)	TA100 (S9−)	TA100 (S9+)
		Mean ± SD			
Negative Control		45 ± 13	45 ± 5	105.3 ± 5	99 ± 1.7
Positive control		1206	1508	1905	2132
Imazalil	20.4 μg/plate	65 ± 8.7	84.3 ± 6.6 *	129.6 ± 5.8 *	124 ± 10.7
Imazalil	40.8 μg/plate	53 ± 2	111 ± 5.2 *	156.3 ± 8.9 *	138 ± 22.6
Pyrimethanil	15 μg/plate	46.3 ± 16	28 ± 3.6	129.3 ± 7 *	91.6 ± 10
Pyrimethanil	30 μg/plate	48.6 ± 13	29 ± 5.5	111.6 ± 15.9	111.3 ± 4.7 *
Thiabendazole	15 μg/plate	84.6 ± 14 *	40.3 ± 4.7	77.6 ± 19	146.3 ± 2.3 *
Thiabendazole	30 μg/plate	136 ± 29 *	30.6 ± 3.5	120.6 ± 5 *	177.3 ± 46 *
Mixture (IMA + PYR + TBZ)	6.8 + 5 + 5 μg/plate	48.3 ± 6	30 ±8.8	127.5 ± 10.7	97.3 ± 9.3
Mixture (IMA + PYR + TBZ)	13.6 + 10 + 10 μg/plate	52.3 ± 3	29 ± 9.8	122 ± 9.6	98.3 ± 14

* The data were considered significantly different if *p* < 0.05.

**Table 3:** Number of *Salmonella typhimurium* colonies (fungicides in DMSO solvent).

DMSO Samples	Concentration μg/plate	TA98 (S9−)	TA98 (S9+)	TA100 (S9−)	TA100 (S9+)
		Mean ± SD			
Negative control		7.3 ± 1.5	20.3 ± 2.5	85.3 ± 13.4	85.3 ± 13.4
Positive control		2540	762	1143	1016
Imazalil	20.4 μg/plate	30.3 ± 17.03	28.6 ± 8.9	116.6 ± 14 *	112 ±16
Imazalil	40.8 μg/plate	41 ± 22.5 *	10.3 ± 0.5	36.3 ± 9.4	97 ± 15.6
Pyrimethanil	15 μg/plate	15.6 ± 7.2	15.3 ± 2	123 ± 96.2	94.6 ± 16.6
Pyrimethanil	30 μg/plate	28 ± 6 *	15.3 ± 4	71 ± 46.9	92.6 ± 5.8
Thiabendazole	15 μg/plate	21 ± 8.5	96 ± 5.2 *	130.3 ± 8 *	114 ± 9.1 *
Thiabendazole	30 μg/plate	212.6 ± 21.7 *	93.3 ± 7.2 *	81.6 ± 39.1	105 ± 7.9
Mixture (IMA + PYR + TBZ)	6.8 + 5 + 5 μg/plate	16 ± 5.3	21.6 ± 8.4	136.3 ± 12.7	107 ± 3
Mixture (IMA + PYR + TBZ)	13.6 + 10 + 10 μg/plate	11.6 ± 5.03	13.3 ± 1.4	42.6 ± 40.1	74.3 ± 16.9

* The data were considered significantly different if *p* < 0.05.

## Data Availability

The original contributions presented in this study are included in the article. Further inquiries can be directed to the corresponding authors.
